# Knowledge, Practices, and Risk Perception Associated with Foodborne Illnesses among Females Living in Dubai, United Arab Emirates

**DOI:** 10.3390/foods11030290

**Published:** 2022-01-21

**Authors:** Tareq M. Osaili, Balsam Qubais Saeed, Sadi Taha, Ahmed Omar Adrees, Fayeza Hasan

**Affiliations:** 1 Department of Clinical Nutrition and Dietetics, College of Health Sciences, University of Sharjah, Sharjah P.O. Box 27272, United Arab Emirates; 2 Department of Nutrition and Food Technology, Faculty of Agriculture, Jordan University of Science and Technology, Irbid 22110, Jordan; 3 Sharjah Institute for Medical Research, University of Sharjah, Sharjah P.O. Box 27272, United Arab Emirates; U00040620@sharjah.ac.ae; 4 Department of Clinical Sciences, College of Medicine, University of Sharjah, Sharjah P.O. Box 27272, United Arab Emirates; bsaeed@sharjah.ac.ae; 5 School of Business Administration, Al Dar University College, Dubai P.O. Box 35529, United Arab Emirates; sadi@aldar.ac.ae; 6 College of Medicine, University of Sharjah, Sharjah P.O. Box 27272, United Arab Emirates; U18100005@sharjah.ac.ae

**Keywords:** foodborne illnesses, females, cross-contamination, personal hygiene, food storage, food purchase

## Abstract

Foodborne illnesses are a widespread and growing public health concern worldwide. The aim of this study was to investigate the knowledge, practices, and risk perception pertaining to food safety among females living in Dubai, United Arab Emirates (UAE). A questionnaire-based cross-sectional study was undertaken consisting of 827 female participants between January to April 2020. The study showed that the overall food safety risk perception was below satisfactory (53.3%). The highest score was seen in the “recognition of foodborne illnesses” aspect (76.7%). The participants were aware of “personal hygiene and cleaning” (61.7%), “cross-contamination prevention” (62.5%), “food purchasing” (60.0%), and “storage of frozen foods” (55.6%). The participants had a low level of knowledge pertaining to “food cooking” (26.0%) and “risk of microbiological infection” (13.3%). There was a statistically significant (*p* ≤ 0.05) association between knowledge and practices of respondents with employment status, age, and educational levels. In conclusion, the female respondents might act as vehicles for the spread of foodborne illnesses. To reduce this risk, providing food safety awareness programs to this portion of the population is paramount.

## 1. Introduction

Foodborne illnesses are caused due to the consumption of contaminated food or drink. Foodborne outbreaks could result in significant economic losses and are a global public health concern [1,2,3]. According to the World Health Organization (WHO), globally, more than 91 million cases of foodborne outbreaks and 420,000 deaths are associated with foodborne diseases each year [2].

The contamination of food could occur during food preparation, handling, due to improper storage conditions, and lack of personal hygiene [2,4]. The microbiological contaminants causing foodborne illnesses could be bacterial, viral, or parasitic in nature [5]. The most common foodborne pathogens include (but are not limited to) *Escherichia coli*, *Salmonella* spp., *Staphylococcus aureus*, Noroviruses, *Toxoplasma gondii*, *Trichinella spiralis*, and their toxins [6]. The typical clinical presentation of a foodborne illness is usually in the form of gastroenteritis. The severity of the disease varies from case to case; however, in advanced stages, it may result in meningitis, incurable disabilities, and cancer [5]. Considering their substantial impact, countries all over the world are making great efforts to reduce the impact of foodborne outbreaks [7].

Foodborne outbreaks associated with food prepared at homes are usually underestimated because of lower reportage [8]. Studies indicated that around 87% of foodborne illnesses could be attributed to food prepared at home [9,10]. In Arab culture, females usually take care of the kitchen work at home. Many previous studies have observed that the food safety knowledge amongst the females in these countries is low [9,11,12,13,14]. A study conducted in Saudi Arabia indicated 45.3% of the respondents were not aware of the proper food holding temperature [11]. In another study conducted in the region, only 49.8% of the women reported having adequate food safety knowledge scores regarding utensils and equipment [12]. Meanwhile, the mean food safety knowledge score in Jordanian women was 33.9% [14]. A study conducted only in one emirate (Sharjah) of the United Arab Emirates (UAE) indicated only half the respondents were knowledgeable about food safety and followed good practices [13].

Dubai is another emirate in the country. The Food Safety Department of Dubai Municipality stringently imposes internationally accepted comprehensive systems to ensure food safety and disease control at the retail level [15]. However, it has limited command at the domestic level. Thereby, this study aimed to investigate the knowledge and practices pertaining to food safety amongst females living in Dubai (UAE).

## 2. Materials and Methods

### 2.1. Study Design

A cross-sectional study was conducted over the period ranging from 3 January to 4 April 2020. A total of 827 females from different nationalities living in Dubai, UAE, participated in the study. The sample size was calculated based on the formula normally used in cross-sectional studies [16]. The participants exceeded the minimum calculated requirement (*n* = 385).

### 2.2. Questionnaire Design

The questionnaire was adapted from previously conducted studies [9,11,14,17,18]. The first section of the questionnaire was composed of demographic characteristics followed by seven aspects pertaining to food safety knowledge and practices. The demographic characteristics questions included variables pertaining to employment, marital status, age, and education level. The food safety questions could be broadly classified into “food purchase” (4 questions), “storage of frozen foods” (9 questions), “cross-contamination prevention” (4 questions), “personal hygiene and cleaning” (6 questions), “cooking practices” (5 questions), “recognition of foodborne illnesses” (3 questions), and “risk of microbiological infection” (6 questions).

### 2.3. Pilot Study

The questionnaire was formulated in two languages, Arabic and English. To avoid self-reported bias, the questions were revised to ensure that they were clear and short; they took 10–15 min to answer.

The survey was piloted via face-to-face interviews with 15 volunteers to assess the questions’ clarity and appropriateness. Furthermore, the questionnaire was verified by four academic faculty members who acknowledged that the survey questions were clear, appropriate, easy to understand, and measurable. The questionnaire was reliable regarding the overall internal reliability (Cronbach’s Alpha = 0.70).

### 2.4. Sample Collection

The Health Promotion Department at the Supreme Council for Family Affairs aided in disseminating the questionnaire. Women living in the emirate of Dubai were also approached via Facebook and WhatsApp in a snowball fashion. The respondents were informed regarding the objectives of the study and their right to withdraw from the study at any time. Only respondents above the age of 18 were considered eligible to take part in the study. All respondents were asked to sign a consent form prior to the commencement of the questionnaire. All responses were treated in a confidential and anonymous manner.

### 2.5. Statistical Analysis

Statistical analysis was conducted using the IBM Statistical Package for Social Sciences (SPSS version 23, Chicago, IL, USA). Descriptive statistics tests were conducted to analyze the demographic characteristics of the participants. Categorical variables were described in percentages. The knowledge and practices of participants’ responses were measured using “I know”, “I don’t know”, and “Not sure”. A score of (1) was given for every correct response, while a score of (0) was given to a wrong answer, ‘I don’t know’, and ‘Not sure’ responses.

One-way ANOVA using Tukey’s HSD post-hoc test was used to test the association between the food safety score, age, and education level of the participants. An independent–samples t-test was used to test the association between the food safety score, employment, and marital status. A *p*-value < 0.05 was considered to be statistically significant.

## 3. Results and Discussion

### 3.1. Participants Demographic Characteristics

A total of 827 female respondents living in Dubai (UAE) participated in the study (Table 1). The sample consisted of 664 (80.3%) married women and 163 (19.7%) single females. More than half of the females (58.5%) were either employed in the private or governmental sector. The age of the participants ranged from 18–65 years. Less than half of the participants (44.9%) were between the age of 34–51 years. With regards to the education level, 49% had completed a bachelor’s degree, 28.5% possessed a postgraduate degree, and 22.5% had finished elementary or high school.

### 3.2. Overall Score of Food Safety Knowledge and Practices

**Figure 1:**
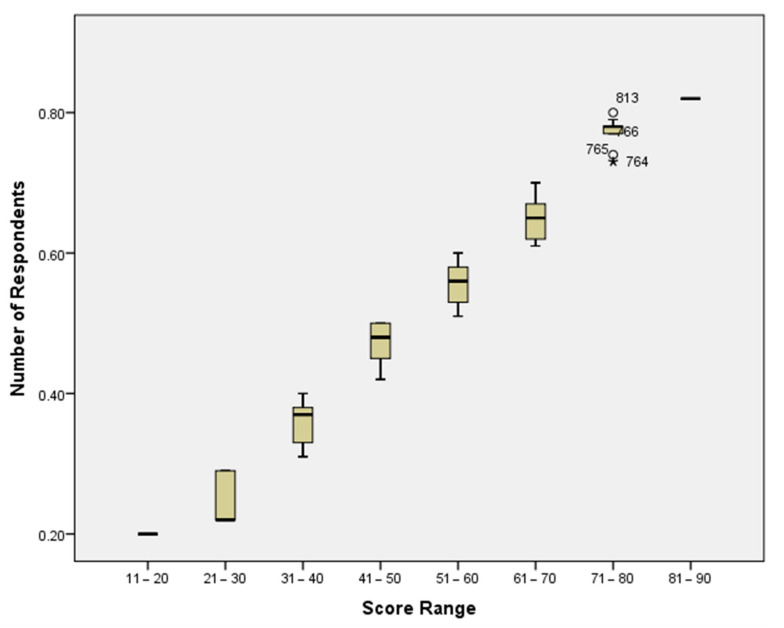
Distribution of food safety knowledge and practices score percentage.

Food safety knowledge and practice scores of the tested seven aspects and the overall score are presented in Table 2. The results indicated that the overall food safety knowledge and practice score among women in Dubai was 19.7 ± 0.1, corresponding to 53%. A box and whisker plot shows the distribution of food safety knowledge and practices score percentage (Figure 1). None of the respondents scored above 90%. The percentage of respondents who scored 21–30, 31–40, 41–50, 51–60, 61–70, 71–80, and 81–90 was 5.2, 13.9, 15.2, 41.7, 16.1, 6.0, and 1.7%, respectively.

The WHO has developed “5 keys”, which explain in detail the various actions through which food safety can be enhanced. The recommendations include general cleanliness, separation of raw and cooked products, thorough cooking, storage of food at safe temperatures, and the use of safe water and raw materials [19]. The questions in this study have also been designed to revolve around this concept.

Overall, two-thirds of the participants in this study exhibited good knowledge and correct practices (score ≥ 60%) in “recognizing foodborne illnesses”, “personal hygiene and cleaning”, “cross-contamination prevention”, and “food purchasing” aspects.

The current study findings were in line with the observations in Egypt [9] and Saudi Arabia [12]. It was observed that participants in this study had low knowledge and poor practices in terms of “cooking practices” and “risk for microbiological infection”. A similar observation was made in Saudi Arabia [12], Sharjah [13], and Italy [20]. These results indicate the need for food safety program development committees in the UAE to give more attention to females at the household level.

#### 3.2.1. Food Purchasing

A score of 2.4 ± 0.3 out of 4.0 (60.0%) was recorded for the “food purchasing” aspect (Table 2). More than half (64%) of the participants knew that the purchase of frozen foods should be towards the end of the shopping trip (Table 3). The current study results were in line with a study conducted in Jordan by Osaili et al. [14], where 74% of female students reported that the safest time to buy refrigerated food was towards the end of shopping time. They were also in accordance with a study by Farahat et al. [12] in Saudi Arabia, where 70.8% of the respondents bought perishable food items towards the end of the shopping trip. Perishable items such as milk and meat need low storage temperature to prevent rapid bacterial growth [21,22].

Although more than half of the participants reported they read the information pertaining to the food product (58.4%), a mere 22.9% read the entire information (Table 3). Reading food labels is recommended as this would result in the consumer paying attention to the expiry dates [23]. A study conducted in Khartoum (Sudan) and Bulgaria reported 77.6 and 50.1% of the respondents had a habit of reading food labels [24,25]. The majority of the respondents (93.2%) stated that they needed less than 1 h to reach home after shopping (Table 3). This is similar to another study where 97.0% of the respondents stated that they reached home within a span of two hours [13].

#### 3.2.2. Storage of Frozen Foods

A score of 5.0 ± 0.2 (55.6%) out of 9.0 was reported for the “storage of frozen foods” aspect (Table 2). Table 4 show that 97.9% of the female respondents in Dubai stored perishable foods in the freezer or refrigerator immediately after arriving home. The majority (79.7%) recorded storing salads and appetizers in the fridge after preparation. For leftovers, 71.6% of the respondents recorded immediate storage in the refrigerator. A survey conducted on women in Riyadh, Saudi Arabia, showed a similar finding where 96.4% of the respondents placed perishable foods in the fridge or freezers immediately after arrival [11]. About 71% kept salad immediately in the fridge after preparation, while another 75.6% transferred leftovers immediately to the fridge [11]. In another study conducted on Italian women, only 49.9% reported keeping leftovers in the refrigerator immediately after meals [20].

Awareness regarding appropriate freezing and refrigeration temperatures is crucial in reducing the risk of food poisoning. In our study, more than 75% of the females did not know the correct operating temperatures of the refrigerator and freezer. Upon being enquired about the effect of fridge temperature on microbial growth, less than half (43%) answered that it slows down the reproduction of microorganisms. These results were similar to the observation of Alsayeqh et al. [11], where only 34.5 and 12.3% of females in Saudi Arabia knew the correct operating temperatures of the refrigerator and freezer, respectively. Another study conducted by Osaili et al. [14] reported that only 34 and 21% of the female university students in Jordan possessed the correct knowledge pertaining to the operating temperatures of refrigerators and freezers, respectively. This lack of knowledge is concerning as the climate in the UAE is extremely hot (with temperatures reaching up to 56°C in the summer). Not knowing/keeping a check on the correct operating temperatures of the freezer and refrigerator could result in rapid food spoilage and microbial growth. Almost half of our respondents wrongly stated that cold temperatures could kill microbes in food. In a study conducted by Alsayeqh [11], 18.5% of the respondents in Saudi Arabia agreed to the same.

The safest thawing method is by keeping the frozen meat/poultry in the fridge was agreed upon by 23.8% of the females. While regarding food storage instructions outside the home (e.g., picnics), the majority of the participants (75%) reported using an icebox to store food. Since the ideal temperature for the growth of pathogenic microorganisms commonly found in food ranges from 21 to 52 °C, meats and other frozen products need to be kept for thawing in the fridge [26]. This is expected to reduce microbial proliferation and toxin production. In a study conducted by Alsayeqh [11], about 23% of women in Saudi Arabia, where climate conditions are very similar to that in UAE, did not possess the knowledge that thawing frozen foods or keeping perishable food outside the fridge could lead to food poisoning.

The females in our study had fairly good knowledge regarding the storage period of an opened bottle of milk. More than half (62.8%) recorded storing opened milk bottle in the fridge and consuming it within 3–4 days after opening. This result was similar to that reported by Alsayeqh [11] for women in Saudi Arabia, where 74.1% of the respondents reported keeping an open bottle of milk in the refrigerator but not being aware of the correct shelf life.

#### 3.2.3. Cross-Contamination Prevention

The food safety knowledge and practice score of the “cross-contamination prevention” aspect among women in Dubai was 2.5 ± 0.2 out of 4.0 (62.5%) (Table 2). As can be seen in Table 5, most of the females (96.5%) recorded having separate sets of cutting boards and knives for vegetables and meat. An excellent percentage (93.1%) recorded washing cutting boards with hot water and soap after every use.

In another study, 54% of the restaurant workers in Jordan reported washing cutting boards were used to cut raw meat or poultry with hot water prior to chopping vegetables [27]. Similarly, 61.6% of female students in a study conducted by Osaili et al. [14] indicated that they washed the cutting board with hot soapy water before chopping the vegetables.

In this study, 64.2% of the women reported storing food in the refrigerator separately as per the type of food and not based on space availability. Storing food separately in the refrigerator is important to avoid cross-contamination [28]. This finding was similar to the observation amongst women in Saudi Arabia (63%) [11] and Trinidad (81.5%) [26].

In this study, 51% of the participants were fully aware that it was best to use single-use paper towels to dry hands after washing them. This practice is important as cloth hand towels may contain different types of enteric bacteria such as *E. coli* and *coliforms* [29]. This knowledge level was higher than that reported amongst women in Alexandria University (14%) [9] and amongst adult consumers in Turkey (10.6%) [30].

#### 3.2.4. Personal Hygiene and Cleaning

The food safety knowledge and practice score of the “personal hygiene and cleaning” aspect was 3.7 ± 0.2 out of 6.0, corresponding to 61.7% (Table 2). Most of the participants (82.2%) reported washing the sink after every use (Table 6). Two-thirds of the respondents (64.1%) knew that a used kitchen sponge would be an ideal breeding ground for bacteria. Less than half (49.2%) reported cleaning kitchen counters with hot water and a disinfectant. To avoid foodborne illnesses, the kitchen sink must be cleaned with hot water, soap, and then a disinfectant after every use [8,31]. The kitchen sponges need to be changed on a regular basis as they could act as a vehicle for cross-contamination [32]. Unclean kitchen sponges have been reported to contain a high number of pathogenic bacteria such as *Campylobacter* spp., *Enterobacter cloacae*, *E. coli*, *Klebsiella* spp., *Proteus* spp., *Salmonella* spp., *Acinetobacter*, *Moraxella*, and *Staphylococcus* spp. [32]. A study conducted on Lebanese food handlers showed that 86.8% of them washed the kitchen sink with hot water, soap, and then a disinfectant [33].

Around 54% of the respondents spent about 20 s or more washing hands with soap and water. More than half (57.9%) knew that washing hands during each stage of the food preparation process is not necessary. Washing hands before preparing food reduces the risk of food poisoning, as contaminated hands of a food handler can spread microbes to food [34]. Our finding is similar to that reported by Jevšnik et al. [35], where 57.1% of the interviewed females had adequate knowledge about correct hand-washing procedures. However, our finding is better than that reported by Fawzi and Shama [9], where only 20% of Egyptian female respondents reported washing hands with warm water and soap. In the current study, 61.2% of the respondents knew that fruits and vegetables should be washed under running tap water. This result was similar to women in Saudi Arabia (63%) [11]. Raw fruits and vegetables could act as vehicles for foodborne pathogenic transmission to humans [36].

#### 3.2.5. Cooking Practices

A food safety knowledge and practice score of 1.3 ± 0.2 out of 5.0, corresponding to 26.0%, was observed in the “cooking practices” aspect (Table 2). Inappropriate cooking temperatures and duration coupled with lack of thermometer measurements can lead to foodborne illnesses [37]. The percentage of females who were deficient in this knowledge in our study (26.0%) was similar to that reported for women from Saudi Arabia (35.5%) [11].

As shown in Table 7, half (50.1%) of the respondents knew the correct way of reheating food. Similarly, in Mainland China and Slovenia, the percentage was reported to be around 52 and 60%, respectively [38,39]. On the other hand, in the current study, a very low percentage of the participants (22.9%) knew that the internal temperature of food should be measured with the help of a thermometer. Only 8.3% of the participants followed the recommended time and temperature for cooking. Previous studies indicated that only 28.4 and 8.2% had knowledge about the use of a thermometer during cooking [11,14]. This study found that 23.6% of the females had the knowledge that heat kills microbes but does not guarantee food safety as some bacterial toxins are resistant to high temperatures [40]. In comparison, a study conducted on Taif University students (Saudi Arabia) showed that 50% knew the effect of heat on microbes [41]. About a quarter (22.9%) of the respondents agreed that raw milk is unsafe to drink. A study conducted by Alsayeqh [11] reported the agreement percentage to be 21.5%, respectively.

#### 3.2.6. Recognition of Foodborne Illnesses

Compared to other segments, the food safety knowledge score recorded in this domain was the highest (2.3 ± 0.3 out of 3.0, 76.7%) (Table 2). As can be seen in Table 8, a good percentage (87.7%) of females knew that diseases could be transmitted through food. The majority (79.9%) of the participants recognized the main symptoms of food poisoning, with more than half (66.1%) having the knowledge that bacteria and viruses were the main causal agents of food poisoning. Foods stored at inappropriate temperatures for long durations or foods handled in an inappropriate manner can result in contamination with bacteria and viruses. These microorganisms can colonize in the human gut and can cause gastrointestinal disturbances such as diarrhea and vomiting. The bacteria may also release toxins that can survive the harsh acidic pH of the stomach. Depending on the type of toxin and the immune status of the consumer, the consequences of the toxin ingestion may lead to fatality. In a previous study, 91.6, 82.0, and 67.8% of the respondents were aware that diseases could be transmitted via food, the various symptoms of food poisoning and the causal agents of food poisoning (bacteria and viruses), respectively [13].

#### 3.2.7. Risk of Microbiological Infection

The knowledge in this segment was less as compared to the other aspects (Table 2). The knowledge score was 0.8 ± 0.2 out of 6.0, corresponding to 13.3%. As can be seen in Table 9, 51.6% knew that food should be cooked well and exposed to heat to be free of *Salmonella* spp. A small percentage (7.9%) knew that *Staphylococcus* bacteria—associated infection could be due to the ingestion of food prepared with the bare hands and then left at room temperature, while only 10.3% were aware that Botulism is associated with canned food. On a similar note, 11.7% of the females knew that *Listeria* infection is associated with hot dogs, white cheeses and meat sandwiches. A similar percentage (12.3%) knew that *E. coli* infection is associated with raw or undercooked beef. A mere 4.0% knew that illnesses due to raw or undercooked poultry are associated with *Campylobacter* spp.

As per the Ministry of Health in Dubai, the most common foodborne diseases reported are amoebic dysentery, bacillary dysentery, typhoid/paratyphoid fever, hepatitis A, giardiasis, shigellosis, and salmonellosis [42]. Poor knowledge of the respondents regarding the most common food associated pathogens was revealed in our findings. A similar observation was reported for Italian consumers where only 56.3% had knowledge regarding foodborne *Salmonella* spp. associated infection [20]. Furthermore, poor knowledge levels were also reported among consumers in Ballsbridge, Dublin, and Ireland, such that only 45.2 and 19.2% of the participants knew the types of food associated with *Salmonella* spp. and *E. coli* infection, respectively [18]. Higher knowledge levels were reported in an Italian study where 86.3% knew information about *Salmonella* spp. and 51.6% had sufficient information on *C. botulinum* [20].

### 3.3. Association between Total Food Safety Knowledge, Practice Score, and Socio-Demographic Characteristics of Females in Dubai, UAE

Table 10 demonstrate the relationship between overall food safety knowledge and practice score of females and their demographical data. In this study, it was observed that a statistical difference existed in the overall food safety knowledge and practice scores of employed and unemployed females (*p*-value < 0.05). Employed women recorded a higher score (20.5 ± 0.1) than unemployed females (18.6 ± 0.1). This is similar to the observations of Farahat et al. [12], where working females showed a higher foods safety knowledge and practice score than non-working women. This study indicated that older and educated females had higher (*p*-value < 0.05) overall knowledge and practice scores. Females holding a postgraduate degree were observed to have a higher knowledge and practice score (20.9 ± 0.1). The age group of 52–65 years was observed to have a higher food safety knowledge and practice score (22.5 ± 0.2) than other examined groups. This is similar to observations in previous studies [12,13,20]. In addition, Ayaz et al. [43] highlighted that knowledge and practices among Saudi Arabia women were improved with the level of education. No statistically significant difference in the score (*p*-value ≥ 0.05) existed between married and single females (Table 10), similar to the study conducted by Osaili et al. [44].

## 4. Conclusions

In this study, a low level of food safety knowledge and inadequate correct practice regarding food safety was observed amongst women in Dubai (UAE). Educating females regarding appropriate food handling/cooking practices and engaging them in food safety workshops is essential. Tools such as the WHO “5 keys”, which give illustrative explanations about cleanliness, the prevention of cross-contamination, and temperature use, can be used for educational purposes.

## Figures and Tables

**Table 1:** Descriptive characteristics of the participating females in Dubai, UAE (*n* = 827).

Demographic Variables		Frequency	Percentage %
Marital status	Married	664	80.3
	Single	163	19.7
Employment	Employed	484	58.5
	Unemployed	343	41.5
Age	18–24 years	108	13.1
	25–33 years	220	26.6
	34–51 years	371	44.9
	52–65 years	128	15.5
Education	Primary	70	8.5
	High school/Diploma	116	14.0
	Undergraduate	405	49.0
	Postgraduate	236	28.5

**Table 2:** Food safety knowledge and practices score (Mean ± SD) and percentages of correct answers of females in Dubai, UAE.

Food Safety Aspects	Mean Knowledge Scores (SD)	Possible Range of Scores	Percentage of Correct Responses
(1) Food purchasing	2.4 ± 0.3	0–4	60.0
(2) Storage of frozen foods	5. 0± 0.2	0–9	55.6
(3) Cross-contamination prevention	2.5 ± 0.2	0–4	62.5
(4) Personal hygiene and cleaning	3.7 ± 0.2	0–6	61.7
(5) Cooking practices	1.3 ± 0.2	0–5	26.0
(6) Recognizing of foodborne illness	2.3 ± 0.3	0–3	76.7
(7) Risk of microbiological infection	0.8 ± 0.2	0–6	13.3
Total knowledge score	19.7 ± 0.1	0–37	53.2

**Table 3:** Correct responses * to the “Food purchasing” aspect of females in Dubai, UAE.

Food Safety Aspects	Total Responses (*n*)	Correct Responses (%)
(1) During shopping, when do you purchase refrigerated and cold foods? **At the end of shopping time**	529	64.0
(2) Do you read the label on food products during shopping? **Yes**	483	58.4
(3) What do you read on food labeling? **All information: storage instructions; ingredients; expiry date.**	189	22.9
(4) (How much time does it take to return home after shopping? **Less than 1 h**	771	93.2

* Statement in bold color is the correct answer.

**Table 4:** Correct responses * to the “Storage of frozen foods” aspect of females in Dubai, UAE.

Food Safety Aspects	Total Responses (*n*)	Correct Responses (%)
(1) Where do you store meat, poultry, or fish after arriving home? **Put it immediately in refrigerator or freezer**	810	97.9
(2) What do you do with salads or appetizers after preparation? **Put them immediately in the refrigerator**	659	79.7
(3) What do you do with food leftover? **Put it immediately in the refrigerator**	592	71.6
(4) The appropriate refrigerator temperature is? **1–4 °C**	207	25.0
(5) The appropriate freezer temperature is? **At least −18 °C**	178	21.5
(6) What is the effect of the cold temperature of the fridge on microbes? **Delays microbial growth in food**	356	43.0
(7) How do you defrost frozen meat or poultry? **Keep it in the refrigerator**	197	23.8
(8) Do you use your icebox (cooler) when you go for a picnic? **Yes**	620	75.0
(9) What do you do with opened milk bottle? **Keep in the refrigerator and consume within three days**	519	62.8

* Statement in bold color is the correct answer.

**Table 5:** Correct responses * to the “Cross-contamination prevention” aspect of females in Dubai, UAE.

Food Safety Aspects	Total Responses (*n*)	Correct Responses (%)
(1) What would you do if you wanted to cut vegetables after cutting raw meat or poultry? **Use a cutting board and knives set for meat and another set for vegetables**	798	96.5
(2) When should the cutting board and knives be washed? **After every use**	770	93.1
(3) How do you store foods in the refrigerator? **Separate foods according to type**	531	64.2
(4) How would you dry your hands? **Disposable tissues**	421	50.9

* Statement in bold color is the correct answer.

**Table 6:** Correct responses * to the “Personal hygiene and cleaning” aspect of females in Dubai, UAE.

Food Safety Aspects	Total Responses (*n*)	Correct Responses (%)
(1) When should the kitchen sink be washed? **After every use**	680	82.2
(2) Are kitchen sponges contaminated with a high number of bacteria? **Yes**	530	64.1
(3) Should kitchen counters be washed with hot water, soap then disinfected? **Yes**	407	49.2
(4) For how long should hand-washing with soap and water last? **20 s or more**	448	54.2
(5) On which of the following occasions, hand washing is not necessary? **After every stage of food preparation**	479	57.9
(6) How do you wash vegetables and fruits? **With tap water**	506	61.2

* Statement in bold color is the correct answer.

**Table 7:** Correct responses * to “Food Cooking” aspect of females in Dubai, UAE.

Food Safety Aspects	Total Responses (*n*)	Correct Responses (%)
(1) How do you heat food leftover? **Heat it until boiling**	414	50.1
(2) Is using a thermometer is the safest way to know if meat is cooked enough: **Yes**	189	22.9
(3) How do you know if food is cooked enough? **By cooking according to the recommended time and temperature**	69	8.3
(4) What is the impact of heat on microorganisms? **Kills** microorganisms but does not guarantee food safety	195	23.6
(5) Is it safe to drink raw milk? **No**	189	22.9

* Statement in bold color is the correct answer.

**Table 8:** Correct responses * to the “Recognizing of foodborne illness” aspect of females in Dubai, UAE.

Food Safety Aspects	Total Responses (*n*)	Correct Responses (%)
(1) Do you think that diseases can be transmitted through food? **Yes**	725	87.7
(2) Identify which one of the following is not a common symptom of food poisoning: **Joint pain**	661	79.9
(3) Are bacteria and viruses the main cause of food poisoning: **Yes**	547	66.1

* Statement in bold color is the correct answer.

**Table 9:** Correct responses * to the “Risk for microbiological infection” aspect of females in Dubai, UAE.

Food Safety Aspects	Total Responses (*n*)	Correct Responses (%)
(1) *Salmonella* bacteria can cause food poisoning. How can a food be made safe if it has *Salmonella* in it? **Cook it thoroughly**	427	51.6
(2) Staph (*Staphylococcus*) bacteria are most likely associated with which food? **Food prepared with the bare hands and then left at room temperature**	65	7.9
(3) Botulism is a disease that is most likely associated with which food? **Canned foods**	85	10.3
(4) *Listeria* bacteria are most likely associated with which food? **Deli meats, hot dogs and white cheeses**	97	11.7
(5) Harmful *E. coli* bacteria are most likely associated with which food? **Raw or undercooked beef**	102	12.3
(6) *Campylobacter* bacteria are most likely associated with which food? **Raw or undercooked poultry**	33	4.0

* Statement in bold color is the correct answer.

**Table 10:** Association between overall food safety knowledge and practices score and descriptive characteristics of females in Dubai, UAE.

Variable	Total Knowledge Score (Mean)	Total Knowledge Score (%)	*p*-Value
**Employment**			
Employed	20.5 ^a^	55.4	0.000 *
Unemployed	0.18 ^b^	55.2	
**Education**			
Primary	12.1 ^b^	32.7	0.000 *
High school/Diploma	20.0 ^a^	54.0	
Undergraduate	20.2 ^a^	54.7	
Postgraduate	20.9 ^a^	56.4	
**Age**			
18–24 years	18.5 ^b^	49.9	0.000 *
25–33 years	19.7 ^b^	53.4	
34–51 years	19.8 ^b^	53.4	
52–65 years	22.5 ^a^	60.7	
**Marital Status**			
Married	19.5	52.8	0.059
Single	20.3	55.0	

* Means with different letters in the same column are significantly different at <0.05.

## Data Availability

The data presented in this study are available on request from the corresponding author.
